# COVID-19 Mortality and Progress Toward Vaccinating Older Adults — World Health Organization, Worldwide, 2020–2022

**DOI:** 10.15585/mmwr.mm7205a1

**Published:** 2023-02-03

**Authors:** Man Kai Wong, Donald J. Brooks, Juniorcaius Ikejezie, Marta Gacic-Dobo, Laure Dumolard, Yoann Nedelec, Claudia Steulet, Zyleen Kassamali, Ayse Acma, Brian N. Ajong, Sandra Adele, Maya Allan, Homa Attar Cohen, Adedoyin Awofisayo-Okuyelu, Finlay Campbell, Veronica Cristea, Stephane De Barros, Ntokwo Vabi Edward, Aura R. Escobar Corado Waeber, Tondri N. Guinko, Henry Laurenson-Schafer, Mostafa Mahran, Raquel Medialdea Carrera, Samuel Mesfin, Emily Meyer, Alessandro Miglietta, Bernadette B. Mirembe, Maribeth Mitri, Ingrid Hammermeister Nezu, Stephanie Ngai, Ojong Ojong Ejoh, Sydel R. Parikh, Emilie Peron, Nikola Sklenovská, Savine Stoitsova, Kazuki Shimizu, Eri Togami, Yeo Won Jin, Boris I. Pavlin, Ryan T. Novak, Olivier Le Polain, James A. Fuller, Abdi Rahman Mahamud, Ann Lindstrand, Bradley S. Hersh, Katherine O’Brien, Maria D. Van Kerkhove

**Affiliations:** ^1^Global Immunization Division, Center for Global Health, CDC; ^2^Department of Immunization, Vaccines and Biologicals, World Health Organization, Geneva, Switzerland; ^3^Health Emergencies Programme, World Health Organization, Geneva, Switzerland; ^4^Office of the Director, Center for Global Health, CDC; ^5^Division of Global Health Protection, Center for Global Health, CDC.

After the emergence of SARS-CoV-2 in late 2019, transmission expanded globally, and on January 30, 2020, COVID-19 was declared a public health emergency of international concern.* Analysis of the early Wuhan, China outbreak (*1*), subsequently confirmed by multiple other studies (*2*,*3*), found that 80% of deaths occurred among persons aged ≥60 years. In anticipation of the time needed for the global vaccine supply to meet all needs, the World Health Organization (WHO) published the Strategic Advisory Group of Experts on Immunization (SAGE) Values Framework and a roadmap for prioritizing use of COVID-19 vaccines in late 2020 (*4*,*5*), followed by a strategy brief to outline urgent actions in October 2021.^†^ WHO described the general principles, objectives, and priorities needed to support country planning of vaccine rollout to minimize severe disease and death. A July 2022 update to the strategy brief^§^ prioritized vaccination of populations at increased risk, including older adults,^¶^ with the goal of 100% coverage with a complete COVID-19 vaccination series** for at-risk populations. Using available public data on COVID-19 mortality (reported deaths and model estimates) for 2020 and 2021 and the most recent reported COVID-19 vaccination coverage data from WHO, investigators performed descriptive analyses to examine age-specific mortality and global vaccination rollout among older adults (as defined by each country), stratified by country World Bank income status. Data quality and COVID-19 death reporting frequency varied by data source; however, persons aged ≥60 years accounted for >80% of the overall COVID-19 mortality across all income groups, with upper- and lower-middle–income countries accounting for 80% of the overall estimated excess mortality. Effective COVID-19 vaccines were authorized for use in December 2020, with global supply scaled up sufficiently to meet country needs by late 2021 (*6*). COVID-19 vaccines are safe and highly effective in reducing severe COVID-19, hospitalizations, and mortality (*7**,**8*); nevertheless, country-reported median completed primary series coverage among adults aged ≥60 years only reached 76% by the end of 2022, substantially below the WHO goal, especially in middle- and low-income countries. Increased efforts are needed to increase primary series and booster dose coverage among all older adults as recommended by WHO and national health authorities.

Comparative analysis of COVID-19 deaths and mortality rates by age group during 2020–2021 was conducted using three publicly available WHO data sources: 1) daily country-specific number of reported cases and deaths (aggregate surveillance); 2) weekly country-specific age, sex, and health care worker status of disaggregated cases and deaths (detailed reporting)^††^; and 3) WHO-modeled COVID-19 excess mortality estimates^§§^ (*9*). Because the quality of reported data on COVID-19 deaths appeared to vary among countries by income group, the country-specific ratio of excess mortality estimates to total aggregate reported deaths was mapped geographically to reflect the difference in number of deaths by data source. Because the excess mortality model included all countries and accounted for variability in death reporting, it was used to examine mortality rate^¶¶^ and relative risk for persons aged ≥60 years, stratified by World Bank income group (high, upper-middle, lower-middle, and low), independent of potential data quality differences. Percent coverage with a completed COVID-19 vaccination series for the overall population and for older adults were drawn from reporting countries through the WHO electronic Joint Reporting Form COVID-19 module and WHO Regional Office reporting systems. The definition of older adult varied by country; therefore, older adult vaccination coverage was calculated using each country’s definition and dividing the doses reported administered to those older adults by the United Nation’s Population Division age-specific population figures. The 40 countries that did not report vaccination coverage for older adults in 2021 and 2022 were excluded from analysis. WHO data were accessed through the COVID-19 Vaccine Delivery Partnership Information Hub.***^,^^†††^ Data were analyzed and visualized using R statistical software (version 4.1.1; The R Foundation). This activity was reviewed by CDC and was conducted consistent with applicable federal law and CDC policy.^§§§^

During January 2020–December 2021, daily aggregate surveillance and weekly detailed reporting recorded 5.4 million and 2.5 million COVID-19–associated deaths, respectively; the WHO model estimated 14.9 million excess deaths (Table). COVID-19 mortality rates increased markedly in older age groups: persons aged ≥60 years accounted for 80% of COVID-19–associated deaths reported through weekly detailed surveillance and 82% of estimated deaths from the WHO excess mortality model. Among 73% of low-income countries and 31% of lower-middle–income countries (mostly in the WHO African, Eastern Mediterranean, and European regions), the estimated excess mortality exceeded the total reported deaths through aggregate surveillance by more than tenfold, whereas this difference was less than twofold in most higher-income countries (Figure 1). Despite the difference in reporting completeness by income group, cumulative deaths and mortality were higher among older age groups in all income groups. Upper- and lower-middle–income countries accounted for 81% of the global excess mortality among persons aged ≥60 years^¶¶¶^ (Table). Lower-middle–income countries accounted for 52% of excess deaths worldwide among persons aged ≥60 years, with an annual excess mortality rate of 1,039 per 100,000 persons.

**TABLE:** Reported and estimated excess COVID-19–associated deaths, by age group and COVID-19–associated deaths for persons aged ≥ 60 years by World Bank income groups — worldwide, 2020–2021

Characteristic	Data source (no. of countries)					
	Daily aggregate surveillance (194)		Weekly detailed surveillance (147)*		Excess mortality model (194)	
	Cumulative no. of deaths	Mortality rate^†^	Cumulative no. of deaths (% of total)	Mortality rate^†^	Cumulative estimated deaths (% of total)	Estimated excess mortality rate^§^
Age group, yrs						
0–39	No age disaggregated data		114,179 (5)	1	5,393 (<1)	<1
40–49			123,713 (5)	6	658,114 (4)	34
50–59			265,046 (11)	16	1,988,989 (13)	119
60–69			478,599 (19)	40	4,097,508 (27)	345
70–79			614,762 (25)	96	4,145,706 (28)	654
≥80			889,647 (36)	303	4,014,500 (27)	1,365
**Total for all ages**	**5,430,652**	**35**	**2,485,946**	**16**	**14,910,210**	**96**
**Persons aged ≥60 years**						
**World Bank income group^¶^ (no. of countries)**						
High (58)	No age disaggregated data		1,077,394 (54)	183	1,815,098 (15)	308
Upper-middle (55)			757,692 (38)	89	3,581,994 (29)	423
Lower-middle (45)			145,689 (7)	24	6,340,756 (52)	1,039
Low (34)			859 (<1)	1	519,895 (4)	752
**Total for persons aged ≥60 years**			**1,981,634 **	**94**	**12,257,743 **	**579**

* Only 147 countries submitted a detailed weekly report at least once during the 2-year period. Average reporting frequency by income groups was 86% for high-income, 66% for upper-middle–income, 38% for lower-middle–income, and 28% for low-income countries, out of 104 total expected weekly reports.

^†^ Deaths per 100,000 persons per year.

^§^ Excess deaths per 100,000 persons per year.

^¶^ Two countries do not have World Bank income status (Niue and Cook Islands). https://datahelpdesk.worldbank.org/knowledgebase/articles/906519-world-bank-country-and-lending-groups

**FIGURE 1:**
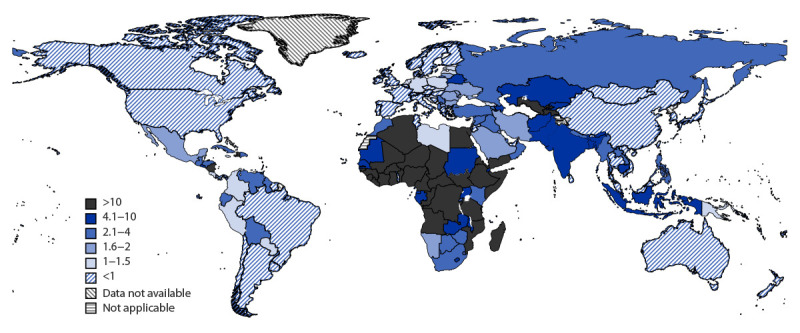
Ratio of excess COVID-19 mortality estimates to aggregate number of reported deaths* — World Health Organization, worldwide, 2020–2021 * The ratio of estimated excess mortality to aggregate reported deaths identified the proportion of deaths that were potentially underreported by countries because of limited testing or nonreporting of causes of death. Model-estimated excess mortality was used in the comparison because it represents a more objective and comparable measure for COVID-19 mortality. Higher ratios represent larger disparities between reported and estimated deaths. https://www.who.int/data/stories/global-excess-deaths-associated-with-covid-19-january-2020-december-2021

As of December 2022, among 194 countries, 154 (79%) had reported both overall and older adult COVID-19 vaccination coverage at least once to WHO; ≥78% of countries in all income groups reported coverage during the last three months of 2022.**** The median overall completed primary series COVID-19 vaccination coverage was 59%, ranging from a low of 21% (low-income countries) (50% [upper-middle–income] and 51% [lower-middle–income]) to a high of 74% (high-income countries). Only high-income countries’ median coverage surpassed the global target of 70% for the overall population. Among older adults, the median completed primary COVID-19 vaccination series coverage was 76%, ranging from 33% (low-income countries) to 90% (high-income countries). Median coverage among older adults in lower-middle– and upper-middle–income countries was 73% and 70%, respectively. Reported coverage among both the overall population and among older adults varied among countries within and among different income groups (Figure 2). Coverage among older adults was the same or lower than that in the overall population in 36 (23%) countries, including four high-income, eight upper-middle–income, 14 lower-middle–income, eight low-income, and two nonclassified countries.^††††^

**FIGURE 2:**
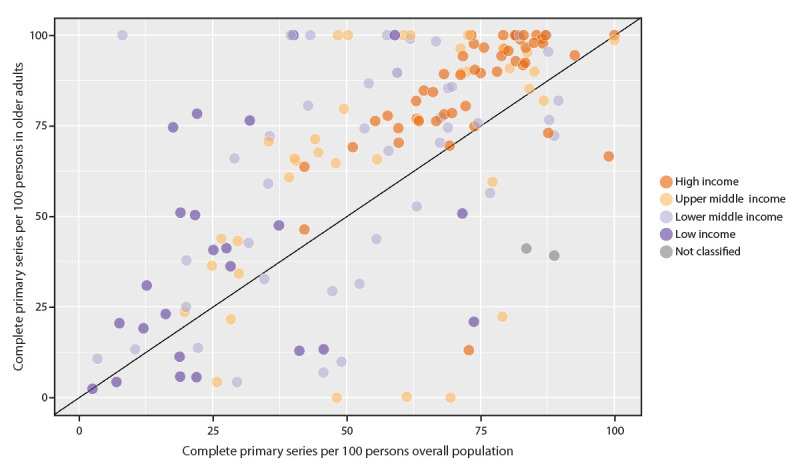
Completed COVID-19 primary vaccination series coverage* reported by countries^†^ among overall population and among older adults, by World Bank income group — World Health Organization, worldwide, December 30, 2022 * Figure shows coverage among older adults was the same or lower than that in the overall population in 36 (23%) countries, including four high-income, eight upper-middle–income, 14 lower-middle–income, eight low-income, and two nonclassified countries. ^† ^The proportions of countries that reported vaccination coverage for older adults at least once during the 2-year period is 83% (48) for the high-income group, 69% (38) for the upper-middle–income group, 96% (43) for the lower-middle–income group, and 68% (23) for the low-income group.

## Discussion

The impact of COVID-19 during the last 3 years has been substantial, and COVID-19 mortality is an important outcome indicator for monitoring the pandemic. These COVID-19 deaths and excess mortality estimates showing that persons aged ≥60 years accounted for more than 80% of total COVID-19 deaths even when controlling for income levels are consistent with the initial SARS-CoV-2–related mortality patterns described in China and subsequently by other countries (*1*–*3*). The large disparity observed between reported deaths and estimated excess mortality, especially in upper-middle–, lower-middle–, and low-income countries, makes ascertaining true COVID-19–associated mortality challenging. Given the bias in reported numbers of age-disaggregated COVID-19 deaths by income group, excess mortality estimates based on modeling might provide a more accurate measure of the impact of the pandemic.^§§§§^ The modeled estimates accounted for limited testing and country-specific reporting of causes of death, particularly as many low- and middle-income countries were known to have faced higher numbers of COVID-19–associated deaths because of insufficient health care capacity. Based on the modeled estimates, persons aged ≥60 years in lower-middle–income countries accounted for more than one half of the global estimated COVID-19 mortality and experienced the highest mortality rate.

COVID-19 vaccines, which have received WHO Emergency Use Listing,^¶¶¶¶^ were introduced in December 2020, <1 year after the first COVID-19 cases were reported. These vaccines have been found to be safe and highly effective in reducing severe COVID-19, hospitalizations, and mortality; however, despite available evidence on effectiveness (*7**,**8*), reported COVID-19 vaccination coverage among older adults has not yet come close to the WHO goal of 100% in many parts of the world. Approximately one quarter of countries reported lower coverage among older adults compared with that in the overall population; many of these countries are in the upper- and lower-middle–income groups, with the highest mortality estimated by the excess mortality model. Further, because of limitations in access to vaccines in many low- and middle-income countries and limited capacity to rapidly roll out COVID-19 vaccines, middle- and low-income countries are taking longer to reach the recommended targets for primary series and booster dose coverage as recommended by WHO and national health authorities.***** As the fourth year of the pandemic begins, vaccine booster doses have been shown to restore or enhance protection against infection, symptomatic disease, and severe disease, beyond that originally afforded by the primary series (*10*). This is particularly important because most countries have ended most mandated public health and behavioral measures to mitigate the spread of SARS-CoV-2.

The findings in this report are subject to at least two limitations. First, age-disaggregated mortality and vaccination data were self-reported by countries with different reporting frequencies, limited data verification processes, and varying capacity for reporting up-to-date information. As a result, reported vaccination coverage rates included in the analysis might be lower than the countries’ published figures because of nonreporting to WHO. Second, the WHO-modeled excess mortality data included all countries and age groups available for 2020 and 2021, but not yet for 2022. Thus, direct association between COVID-19 mortality and vaccination coverage among older adults was not examined in this analysis. Future analyses could be conducted with more regular reports and more availability of detailed data, including vaccination status for reported deaths.

With both timely and reliable data necessary for accurate monitoring of global targets, countries need to be able to strengthen COVID-19 surveillance and vaccination reporting systems to provide better and more detailed data to guide public policies. The collection of accurate disaggregated data by age, and by vaccination dose (i.e., primary series and booster doses), will be critical to monitoring progress in achieving coverage targets among older adults at highest risk for COVID-19–associated death. Because vaccination rates among older adult populations remain below the recommended global vaccination target of 100%, efforts are needed to understand and address the reasons that target populations are not reached by current vaccination programs, while integrating COVID-19 vaccination into primary care systems to facilitate completion of a primary COVID-19 vaccination series and receipt of booster doses recommended by WHO and national health authorities for all older adults.

**Summary**

**What is already known about this topic?**

COVID-19 vaccines are safe and reduce COVID-19 mortality. The World Health Organization (WHO) recommends that countries prioritize populations at increased risk, e.g., older adults, for COVID-19 vaccination with a goal of 100% coverage with a completed primary series for populations at-risk.

**What is added by this report?**

COVID-19–associated mortality among persons aged ≥60 years exceeded 80% of total COVID-19 mortality in 2020 and 2021 across all income groups; however, the median reported completed primary series coverage among older adults in 2022 was 76%, substantially below the WHO goal, especially in middle- and low-income countries.

**What are the implications for public health practice?**

Efforts are needed to increase COVID-19 primary series and periodic booster dose coverage among older adults as recommended by WHO and national health authorities.

## Footnotes

*
https://www.who.int/news/item/30-01-2020-statement-on-the-second-meeting-of-the-international-health-regulations-(2005)-emergency-committee-regarding-the-outbreak-of-novel-coronavirus-(2019-ncov)

†
https://www.who.int/publications/m/item/strategy-to-achieve-global-covid-19-vaccination-by-mid-2022

§ The strategy brief outlined updated goals, steps, targets, and operational priorities to guide countries, policy makers, civil society, manufacturers, and international organizations in their ongoing efforts through 2022. https://www.who.int/publications/m/item/global-covid-19-vaccination-strategy-in-a-changing-world--july-2022-update

¶ Older adult definitions vary by country, ranging from persons aged ≥45 years to those aged ≥65 years.

** Definition of complete primary series might vary among countries and by vaccine product. National authorities have ultimate authority on scheduling decisions within their jurisdictions; however, WHO makes recommendations for COVID-19 vaccine products that have undergone Emergency Use Listing review. Vaccine fact sheets including these definitions according to WHO recommendations can be found at https://extranet.who.int/pqweb/vaccines/vaccinescovid-19-vaccine-eul-issued.

†† Age groups reported by countries are evenly redistributed to match the age groups used in the mortality model.

§§ COVID-19 excess mortality estimates were derived using a statistical regression model developed in collaboration with the United Nations Department of Economic and Social Affairs and an appointed Technical Advisory Group for COVID-19 Mortality Assessment. Mortality estimates include deaths attributable directly to COVID-19 that were not reported, and deaths indirectly associated with COVID-19 attributable to other causes and diseases. WHO defines excess mortality as the mortality above what would be expected based on the noncrisis mortality rate in the population of interest. https://www.who.int/data/stories/global-excess-deaths-associated-with-covid-19-january-2020-december-2021

¶¶ Mortality rates by World Bank income groups were calculated using United Nations population sizes for the countries in each of those income groups. https://www.who.int/data/sets/global-excess-deaths-associated-with-covid-19-modelled-estimates

***
https://infohub.crd.co

††† Disaggregated data on booster dose coverage remain limited. As a result, these data are not included in the current analysis. Recording booster dose coverage by dose (i.e., first booster and second booster) will be critical to continued monitoring of the COVID-19 vaccine rollout.

§§§ 45 C.F.R. part 46.102(l)(2), 21 C.F.R. part 56; 42 U.S.C. Sect. 241(d); 5 U.S.C. Sect. 552a; 44 U.S.C. Sect. 3501 et seq.

¶¶¶ Approximately 70% of the global population of persons aged ≥60 years live in middle-income countries.

**** Proportions among reporting countries that reported COVID-19 primary vaccination series completion among older adults in the last 3 months of 2022 (October–December 2022) were 90% (high-income), 79% (upper-middle–income), 86% (lower-middle–income), and 78% (low-income).

†††† Two countries do not have World Bank Income status (Niue and Cook Islands). https://datahelpdesk.worldbank.org/knowledgebase/articles/906519-world-bank-country-and-lending-groups

§§§§
https://www.who.int/data/stories/the-true-death-toll-of-covid-19-estimating-global-excess-mortality

¶¶¶¶
https://extranet.who.int/pqweb/vaccines/vaccinescovid-19-vaccine-eul-issued

***** https://www.who.int/publications/m/item/covid-19-vaccine-delivery-partnership-september-2022
